# The Influence of Dietary n-3 Highly Unsaturated Fatty Acids on Growth, Fatty Acid Profile, Lipid Metabolism, Inflammatory Response, and Intestinal Microflora in F_2_ Generation Female Yangtze Sturgeon (*Acipenser dabryanus*)

**DOI:** 10.3390/ani14233523

**Published:** 2024-12-05

**Authors:** Jinping Wu, Yuan Liu, Yuqi Wang, Peng Fu, Jiang Luo, Pengcheng Li, Rui Ruan, Junlin Yang, Shijian Xu, Ming Li, Hao Du, Chuang Zhang, Luoxin Li

**Affiliations:** 1 Yangtze River Fisheries Research Institute, Chinese Academy of Fishery Sciences, Wuhan 430223, China; wujinping@yfi.ac.cn (J.W.); luojiang@yfi.ac.cn (J.L.); duhao@yfi.ac.cn (H.D.); 2 Chongqing Fishery Sciences Research Institute, Chongqing 400020, China; 3 Quzhou Sturgeon Aquatic Food Science and Technology Development Co., Ltd., Quzhou 324002, China; 4 College of Animal Science and Technology, Henan Agricultural University, Zhengzhou 450046, China

**Keywords:** Yangtze sturgeon, dietary n-3 HUFA levels, growth, lipid metabolism, inflammatory factors, intestinal microflora

## Abstract

DHA and EPA, as indispensable n-3 highly unsaturated fatty acids (HUFAs), exert a fundamental influence on regulating fish growth, lipid metabolism, and overall well-being. However, there is a notable lack of data concerning their effects on the F_2_ female generation of Yangtze sturgeon. Over a ten-month period, this study assessed the impacts of various dietary concentrations of n-3 HUFAs (0.5%, 1.0%, 1.5%, 2.0%, and 2.4%) on growth, fatty acid composition, lipid metabolism, inflammatory response, and intestinal microbiota in the F_2_ female generation of Yangtze sturgeon. Seventy-five test fish, with an average body weight of 3.60 ± 0.83 kg, were housed in 15 ponds, with each dietary group being assigned to three ponds. The results indicated that the 1.0%~1.5% n-3 HUFA group was characterized by the highest values of weight gain rate; serum triglyceride levels peaked in the 0.5% n-3 HUFA group. The fatty acid profiles of the fish tissues closely mirrored those of the diets. Specifically, compared to the 1.5% and 2.0% n-3 HUFA groups, the diet containing 2.4% n-3 HUFA down-regulated the mRNA expression of transforming growth factor beta, and, compared to the 0.5% and 1.0% n-3 HUFA groups, the 2.0% n-3 HUFA diet up-regulated the mRNA expression of nuclear factor kappa B. Conversely, compared to the 0.5% n-3 HUFA group, 2.0% n-3 HUFA in the diet up-regulated the gene mRNA expression of fatty acid binding protein 1 and fatty acid synthase. Compared to the 0.5% n-3 HUFA group, 1.0% n-3 HUFA in the diet up-regulated the gene mRNA expression of lipoprotein lipase. The α-diversity indices (ACE, PD_whole tree, Richness, and Chao1) exhibited an upward trend with increasing dietary n-3 HUFA levels, and the 2.4% n-3 HUFA group reached the highest values. At the phylum level, Fusobacteriota, Proteobacteria, Firmicutes, and Bacteroidota were the primary dominant phyla. *Cetobacterium* was the dominant genus in all groups. Collectively, these findings underscore that moderate dietary supplementation of n-3 HUFA (1.3%) is optimal and does not impair growth. The deposition of fatty acids in muscle and ovarian tissues, as well as the mRNA expression of lipid-metabolism genes, are closely associated with the dietary n-3 HUFA content. High levels of n-3 HUFA did not suppress intestinal α-diversity. These discoveries provide novel insights into the regulation of growth, lipid metabolism, and health in the F_2_ female generation of Yangtze sturgeon and offer a nutritional strategy for the artificial conservation of this endangered species.

## Simple Summary

Five groups of Yangtze sturgeon were fed diets containing varying levels of high unsaturated fatty acids (HUFAs); each fish had an initial weight of 3.60 ± 0.83 kg and was fed for a duration of 10 months. The objective of this study was to investigate the impacts of these diets on growth performance, fatty acid composition, lipid metabolism, inflammatory factors, and intestinal microbial composition. The findings revealed that the group receiving a diet with 1.0% to 1.5% n-3 HUFA exhibited the highest weight gain rate. Conversely, the group fed a diet containing 0.5% n-3 HUFA had the highest triglyceride levels. Notably, the fatty acid composition of the fish bodies mirrored those of their respective diets. Additionally, specific inflammation-related and lipid-metabolism genes were significantly regulated by n-3 HUFA. Across all groups, *Cetobacterium* emerged as the dominant genus in the intestinal microbiota. Based on the polyline model, incorporating 1.3% n-3 HUFA into the diet was found to be most conducive to weight gain. These results offer novel insights into the regulation of growth, lipid metabolism, and overall health in F2 female Yangtze sturgeon, and provide valuable nutritional strategies for the artificial conservation of this endangered species.

## 1. Introduction

Sturgeon is a chondrostean fish that belongs to the Chordata, Osteichthyes, Actinopterygii, Acipenseriformes, and Acipenseridae. In total, there are 27 species, four of which have become extinct. The remaining 23 species are classified into four genera: two species of *Huso*, two species of *Scaphirhynchus*, three species of *Pseudoscaphirhynchus*, and sixteen species of *Acipenser* [1,2]. China has eight species of sturgeon, including three species that inhabit the Yangtze River: the Chinese paddlefish, *Psephuyrus gladius* (declared extinct) [3]; the Yangtze sturgeon, *A. dabryanus* (declared extinct in the wild in July 2022) [4]; and the Chinese sturgeon, *A*. *sinensis*, which has not reproduced naturally for seven years (2017–2023). In order to save the rare and endangered species of Yangtze sturgeon, Chinese researchers have carried out extensive research with the aim of restoring their artificial populations [5,6,7,8,9,10], but the current results are still not grounds for optimism. As a pure freshwater sturgeon residing in the Yangtze River, the Yangtze sturgeon’s natural reproduction was disrupted in 2000 [11]. Despite achieving the reproduction of its F_2_ generation under conditions of artificial conservation, the cultured individuals encounter a range of issues, including low hatching rate, poorer egg quality compared to wild parents, a high incidence of disease among seedlings, and a low survival rate [12]. The growth, development, and health status of fish are influenced by numerous factors, with nutrition being a pivotal one.

The three major nutrients for fish are protein, lipid, and carbohydrates. Fish meal is the optimal protein source for aquatic animals, but its production is becoming increasingly limited worldwide. Accordingly, lipids and carbohydrates have been extensively utilized as non-protein energy sources in diets, serving as partial substitutes for protein content [13]. However, fish exhibit a low tolerance towards carbohydrates, especially carnivorous fish, and their ability to harness energy from digestible carbohydrates is restricted [14,15]. Consequently, lipids become a more important energy source than carbohydrates in the feeding of carnivorous fish, and have a protein-sparing effect [15]. Lipids fulfil numerous crucial roles, including providing energy and essential fatty acids, serving as a carrier for fat-soluble vitamins, and being a vital component of cell membranes [15,16]. Notably, n-3 HUFA evinces significant functions, including influencing growth, lipid metabolism, and immunity in aquatic animals [17,18,19,20]. Sturgeon, as a sub-cold-water fish, has a higher demand for lipids than do common freshwater fish, highlighting the importance of studying its lipid or highly unsaturated fatty acid requirements during specific life history stages. Currently, numerous reports exist describing highly unsaturated fatty acids studies pertaining to various fish species [17,18,19,20,21,22,23], yet there are a scarcity of data focusing on highly unsaturated fatty acids associated with sturgeon. For instance, several studies exist that have mainly focused on emphases on the crucial regulatory roles of DHA, EPA, and ARA in the early growth, steroid regulation, antioxidant levels, immunity levels, and parental development of sturgeon [24,25,26,27,28]. Additionally, sturgeon is described as a fish species with a large size, late sexual maturity, a long period between spawning, and extended longevity [29], while highlighting the long-term effects of highly unsaturated fatty acids. Thus, we re-fed the test fish previously described in published work in the journal *Antioxidants* (2024, 13, 421) for an additional period of five months. The aim of the current study was to delve deeper into the pivotal role of n-3 HUFA in diet over a longer period, with a focus on its comprehensive regulatory mechanism-based impacts on the growth, lipid metabolism, inflammatory factors, and intestinal microbiota of Yangtze sturgeon. Our ultimate objective was to identify the optimal content of n-3 HUFA for sustaining the healthy growth of Yangtze sturgeon in the long run, thereby providing nutritional regulation strategies for its aquaculture and ensuring safety throughout the entire culture process.

## 2. Materials and Methods

### 2.1. Experimental Diet

In this study, the primary protein sources consisted of fish meal, soybean meal, chicken meal, and squid power. As for the carbohydrate source, it predominantly comprised flour. Additionally, soybean oil, arachidonic acid (ARA) oil, purified docosahexaenoic acid (DHA), and eicosapentaenoic acid (EPA) oil functioned as the lipid sources in the formulation of five groups of experimental diets with equivalent nitrogen and energy content. Refer to Table 1 and Table S1 for the formulations and nutritional compositions. Upon testing, the total EPA and DHA contents in the five groups of diets were found to be 6.18%, 11.45%, 15.53%, 22.17%, and 26.9%, respectively (% of the total fatty acids). Both EPA and DHA oil, which are extracted from fish oil, have concentrations of 78.44% and 98.23%, respectively. The ARA oil derived from the fermentation method using *Mortierella alpina* was utilized in oil production. Its content level was 44.88%. The EPA and DHA oil were sourced from Shanxi Pioneer Biotech Co., Ltd., Shanxi, Hanzhong. The ARA oil was sourced from Fuxing Biotechnology Co., Ltd., Hubei, Hanchuan, China. The feed materials of protein sources and carbohydrate source were obtained from Jinjia feed Co., Ltd., Zhejiang, Hangzhou, China. The blended diet ingredients were processed into long strip diets, each measuring 2 mm in diameter and 20 mm in length, using a meat grinder (MM32, Hunan Hengji Machinery Co., Ltd., Hunan, Yiyang, China). Subsequently, the diets were dried away from light using a fan and preserved in a freezer at −20 °C for future use.

### 2.2. Fish and Feeding Trial

The female Yangtze sturgeon at stage II originated from the Taihu Experimental Station of the Yangtze River Fisheries Research Institute, Chinese Academy of Fishery Science. Before the experiment began, 75 fish that had undergone gender determination were randomly assigned to 15 indoor concrete ponds, each possessing a diameter of 3 m and a depth of 0.5 m. The determination of stage II development primarily relies on ovarian tissue sections. For detailed information regarding gender identification and the tissue sectioning process, refer to the existing report in [28]. During the experiment, the water temperature fluctuated between 15.5 °C and 22.2 °C (mean:19.51 °C). The dissolved oxygen levels ranged from 5.14 mg/L to 9.97 mg/L, averaging 7.44 mg/L. The pH of the water varied between 7.93 and 8.16, with a mean value of 8.07. Additionally, the NH_4_-N concentration fluctuated between 0.04 mg/L and 0.28 mg/L, averaging 0.13 mg/L. Sewage was drained twice daily, and the feeding rate was maintained at approximately 0.8%. The amount of diet was adjusted according to the condition of the test fish and the prevailing weather conditions.

### 2.3. Collection and Analysis of Samples

#### 2.3.1. Analysis of Growth Performance, Serum Biochemistry, and Nutritional Composition

The feeding test encompassed a duration of 10 months. Upon conclusion, the test fish were fasted for a period of 24 h. Subsequently, the fish in each experimental group were individually weighed, and the counts of survivors tallied. From this data, the survival rate and weight gain rate were calculated. During the sampling process, two fish were randomly selected from each breeding pool. These fish were anesthetized with MS-222 (GREENHX Biological Technology Co, Ltd., Beijing, China), enabling the measurement of their body weight and length. These measurements were then used to compute the condition factor. Blood was extracted from the caudal vein using a 5 mL syringe. After allowing the blood to stand in a 4 °C refrigerator for 3 h, it was centrifuged at 4 °C for 10~15 min. The resulting supernatant was collected for further analysis. The test fish were then dissected, with the internal organs carefully excised and weighed. This allowed us to determine the visceral somatic index. Similarly, the liver was separated and weighed to calculate the hepatosomatic index. Furthermore, the hindgut was separated, and the intestinal contents collected for the determination of intestinal microbial composition. Portions of the dorsal muscle, liver, spleen, and ovarian tissues were taken for analysis. One portion was placed in a centrifuge tube containing 4% paraformaldehyde for section preparation, while the other was stored in a −20 °C and −80 °C refrigerator for subsequent analyses of nutrient composition and gene expression.

The following formula was utilized to compute growth performance-related parameters.

Survival rate (SR, %) = (final fish number/initial fish number) *×* 100

Weight gain rate (WGR, %) = (final weight − initial weight)/initial weight *×* 100

Condition factor (CF, %) = 100 × body weight (g)/body length^3^ (cm)

Hepatosomatic index (HSI, 100) = 100 × liver weight (g)/body weight (g)

Viscerosomatic index (VSI, 100) = 100 × viscera weight (g)/body weight (g).

The contents of serum triglycerides (TG), total cholesterol (TC), high-density lipoprotein cholesterol (HDLC), and low-density lipoprotein cholesterol (LDLC) were measured using an automatic biochemical analyzer (BS-460; Shenzhen Mindray Biomedical Electronics Co., Ltd. Guangdong, Shenzhen, China).

The dietary contents of crude protein, crude lipid, and ash were determined using the methods described in AOAC (1995) [30]. Additionally, the moisture contents of the diets were analyzed by oven-drying at 105 °C. The moisture contents of the liver, muscle, and ovary were determined using vacuum freeze-drying for 48 h. The detection of fatty acids in both diets and tissues was conducted according to the methods described by Wu et al. [27]. Muscle and liver samples were sectioned at 8 μm, and stained with oil-red O. The relative area (%) of lipid droplets in oil-red O staining was analyzed by Image-Pro Plus 6.0 [31].

#### 2.3.2. Liver and Spleen mRNA Expression Analysis

RNA samples from the liver and spleen of the Yangtze sturgeon in each group were extracted using the RNeasy Plus Mini Kit (QIAGEN, Hilden, Germany). Total RNA was used to synthesize cDNA by reverse transcription using the PrimerScript RT reagent kit with gDNA Erase (TaKaRa, Shiga, Japan) according to the manufacturer’s instructions. The primers used for qRT-PCR were designed using Oligo software (Version 7) and are shown in Table S2. The qRT-PCR was conducted on a QuantStudio6Flex Real-time PCR system (Thermo Fisher Scientific, Waltham, MA, USA) with β-actin as the reference gene, using the TB Green^®^ Premix Ex Tag” I (TlRNaseH Plus; TaKaRa, Shiga, Japan). Each sample was amplified in triplicate with the following profile: 95 °C for 5 min, and then 95 °C for 30 s, 58 °C for 15 s, and 72 °C for 15 s, for a total of 40 cycles. The relative level of gene expression was analyzed using the 2^−ΔΔCt^ method [32].

#### 2.3.3. Intestinal Microbiota Analysis Procedure

The detection of intestinal microbiota was entrusted to Norminkoda Biotechnology Co., Ltd., Wuhan, China. Microbial community genomic DNA was extracted using the E.Z.N.A.^®^DNA Kit (Omega Bio-tek, Norcross, GA, USA) following the manufacturer’s instructions. The quantity and quality of the extracted DNA were then determined using a NanoDrop 2000 UV-vis spectrophotometer (Thermo Scientific, Wilmington, DE, USA). The hypervariable region V3–V4 of the bacterial 16S rRNA gene was amplified with primer pairs 338F (5′-ACTCCTACGGGAGGCAGCAG-3′) and 806R (5′-GGACTACHVGGGTWTCTAAT-3′) in an ABI GeneAmp^®^ 9700 PCR thermocycler (ABI, Carlsbad, CA, USA). The thermal cycle consisted of an initial denaturation step at 95 °C for 3 min, followed by 27 cycles of denaturing at 95 °C for 30 s, annealing at 55 °C for 30 s, and extension at 72 °C for 45 s. A final extension step was performed at 72 °C for 10 min, with the cycle ending at 4 °C. The PCR components included 4 μL of 5 × TransStart FastPfu buffer, 2 μL of 2.5 mM dNTPs, 0.8 μL of forward primer (5 μM), 0.8 μL of reverse primer (5 μM), 0.4 μL of TransStart FastPfu DNA Polymerase, 10 ng of template DNA, and ddH_2_O to a final volume of 20 μL. The PCR product was extracted from a 2% agarose gel and purified using the AxyPrep DNA Gel Extraction Kit (Axygen Biosciences, Union City, CA, USA) according to the manufacturer’s instructions. The purified product was then quantified using a Quantus™ Fluorometer (Promega, Madison, WI, USA). The purified amplicons were pooled in equimolar portions and subjected to paired-end sequencing on an Illumina MiSeq PE300 platform/NovaSeq PE250 platform (Illumina, San Diego, CA, USA) according to the standard protocols by Wefind Biotechnology Co., Ltd. (Wuhan, China). The raw 16S rRNA gene-sequencing reads were demultiplexed, quality-filtered using fastp version 0.20.0 [33], and merged by FLASH version 1.2.7 [34]. Operational taxonomic units (OTUs) with 97% similarity cutoff [35,36] were clustered using UPARSE version 7.1 [35], and chimeric sequences were identified and removed. The taxonomy of each OTU representative sequence was analyzed by RDP Classifier version 2.2 [37] against the 16S rRNA database using confidence threshold of 0.7. 

### 2.4. Statistical Analysis

Data in the present study are shown as the means ± SE (standard error) and were analyzed using one-way ANOVA after verifying normality and homogeneity using an IBM 22.0 (SPSS, Michigan Avenue, Chicago, IL, USA). When ANOVA-identified overall differences were significant, Tukey’s test was used to compare the mean values among the treatments. A nonparametric test (Kruskal–Wallis) was also used for the comparison, in cases in which the data did not exhibit homogeneity of variance. The significant difference level was set at *p* < 0.05.

## 3. Results

### 3.1. Growth Performance and Morphological Parameters

**Figure 1:**
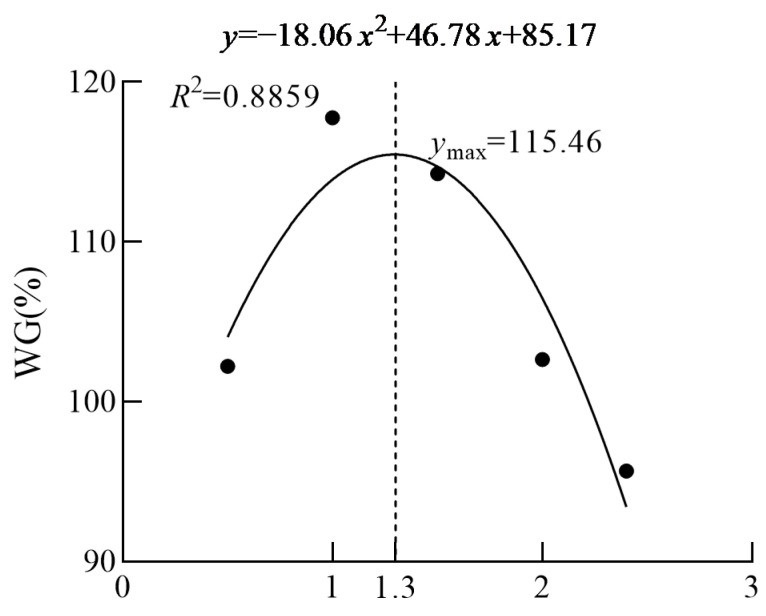
The relationship between weight gain rate and dietary n-3 HUFA level, based on broken-line regression analysis, in which Xopt represents the optimal dietary n-3 HUFA levels for the maximum weight gain rate of Yangtze sturgeon *Acipenser dabryanus*.

The parameters of the WGR, CF, HSI, VSI, and survival rate were not influenced by the dietary n-3 HUFA level (*p* > 0.05) (Table 2). The relationship between weight gain rate and dietary n-3 HUFA levels was described with the broken-line regression model, which indicated that the optimal dietary n-3 HUFA level for attaining maximum growth performance in Yangtze sturgeon *Acipenser dabryanus* was 1.3% of diet. Equations: Y = −18.06*x*^2^ + 46.78*x* + 85.17, *R*^2^ = 0.8859; *x* = 1.3, Y_max_ = 115.46 (Figure 1).

### 3.2. Lipid Components of the Serum 

The serum lipid indices are presented in Table 3. Dietary levels of n-3 HUFA did not significantly impact the serum concentrations of HDL-C, LDL-C, and TCHO (*p* > 0.05). As the levels of n-3 HUFA increased, the serum TG concentrations decreased, with fish fed diets containing 2.0% and 2.4% n-3 HUFA exhibiting significantly lower TG levels compared to those fed the 0.5% n-3 HUFA diet (*p* < 0.05).

### 3.3. Proximate Compositions of the Liver, Muscle, and Ovary

The proximate compositions of liver, muscle, and ovary were not influenced by the dietary n-3 HUFA levels (*p* > 0.05) (Table 4).

### 3.4. Fatty Acid Profiles for the Muscle, Liver, and Ovarian Tissue of F_2_ Generation Female Yangtze Sturgeon Fed Diets Containing Different Levels of n-3 HUFA

The fatty acid profiles of the liver, muscle, and ovarian tissues of the experimental fish are presented in Table 5, Table 6 and Table 7, respectively. In the muscle, the contents of C14:0, C16:0, C18:1n9, and C20:4n-6 were not affected by the dietary n-3 HUFA levels (*p* > 0.05). The contents of C18:0, C18:2n-6, C18:3n-6, C20:2, and C20:3n-6 decreased along with a increase in n-3 HUFA levels, and the contents of these fatty acids in fish fed with the 2.4% n-3 HUFA diets were significantly lower than in the fish fed with the 0.5% n-3 HUFA diet (*p* < 0.05). Contrastingly, the contents of C16:1, C17:0, C20:5n-3, and C22:6n-3 markedly increased with n-3 HUFA levels (*p* < 0.05). The C20:1 content reached its highest levels in the fish fed with the 2.4% n-3 HUFA diet, and these levels were significantly higher than in the other groups (*p* < 0.05). The C18:3n-3 demonstrates a tendency of initially escalating and subsequently decreasing in correlation with the n-3 HUFA content in the diet and reached its highest levels in the fish fed with the 1.5% n-3 HUFA diet; they were significantly higher than in the 0.5%, 1.0%, and 2.4% groups (*p* < 0.05).

In the ovarian samples, the contents of C14:0, C15:0, C17:0, C16:1, C18:1n9, C20:1, C20:2, C20:3n-6, C22:1, and C20:4n-6 were not affected by the dietary n-3 HUFA levels (*p* > 0.05). The contents of C16:0, C18:0, C18:2n-6, C18:3n-6, and C18:3n-3 decreased along with a increase in n-3 HUFA levels, and the contents of these fatty acids in fish fed with the 2.4% n-3 HUFA diet were significantly lower than in the fish fed with the 0.5% n-3 HUFA diet (*p* < 0.05). At the same time, the contents of C20:5n-3 and C22:6n-3 markedly increased with n-3 HUFA levels (*p* < 0.05). The content of C20:3n-3 in fish fed with a 2.4% n-3 HUFA diet was significantly lower than in the fish fed with the 1.0% n-3 HUFA diet (*p* < 0.05).

In the liver, the contents of C14:0, C16:0, C16:1, C18:0, C18:1n9, C18:2n-6, C20:1, C18:3n-3, C20:2, C20:3n-6, C20:4n-6, C20:5n-3, and C22:6n-3 were not affected by the dietary n-3 HUFA levels (*p* > 0.05). The contents of C18:3n-6 decreased along with a increase in n-3 HUFA levels; in fish fed with the 2.4% n-3 HUFA diet, values were significantly lower than in the fish fed with the 0.5% n-3 HUFA diet (*p* < 0.05).

### 3.5. Histological Analysis of the Liver and Muscle

**Figure 2:**
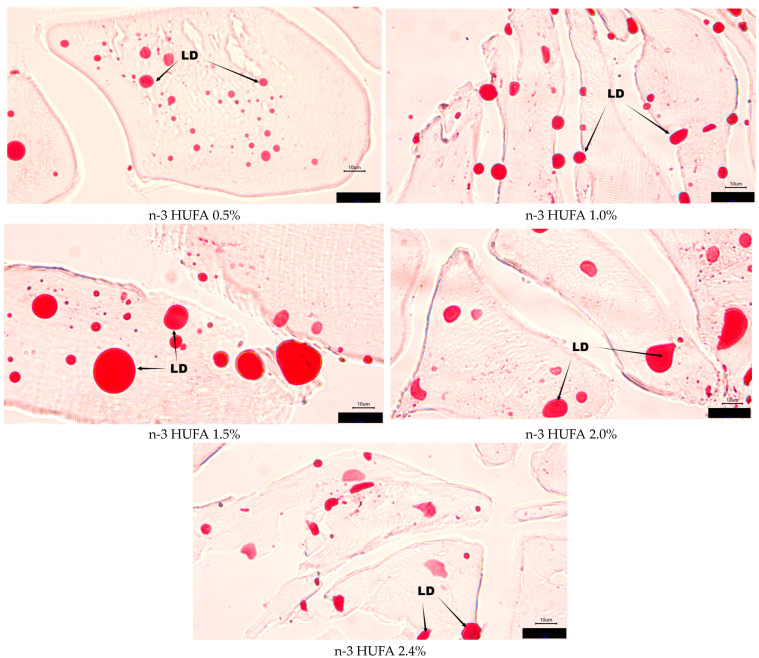
Muscle histology (oil-red O staining, magnification ×100, bars 10 μm). Sample identities are shown in the upper-left corners according to experimental group. LD: lipid droplet.

**Figure 3:**
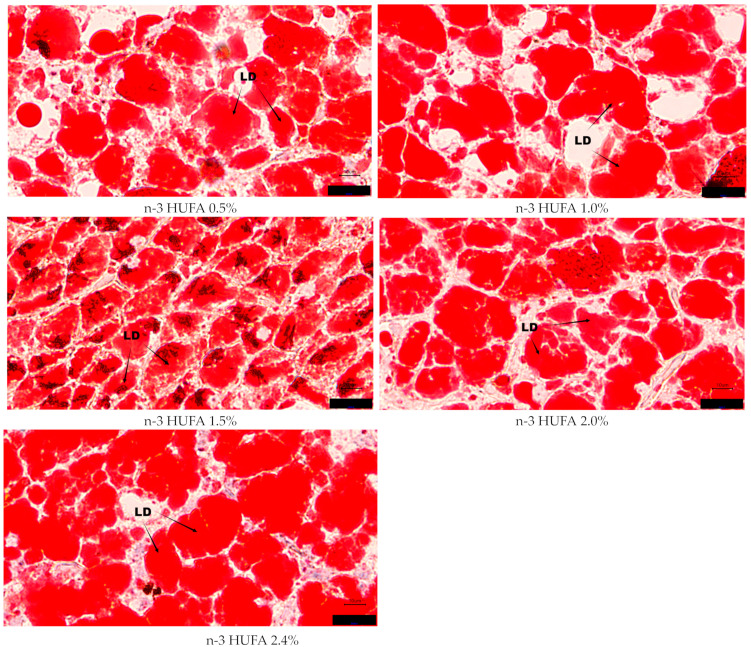
Liver histology (oil-red O staining, magnification ×100, bars 10 μm). Sample identities are shown in the upper-left corners according to experimental group. LD: lipid droplet.

**Figure 4:**
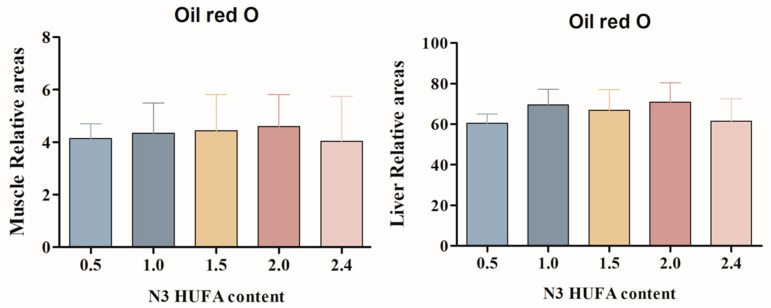
The relative areas (%) of lipid droplets in oil-red O-stained muscles and liver of F_2_ generation female Yangtze sturgeon *Acipenser dabryanus* fed different n-3 HUFA diets. Values are expressed as mean ± SE (n = 6, 6 fish were sampled for each group, and three microscope fields randomly examined for each sample). Different letters indicate significant differences (*p* > 0.05).

The histology images of muscle and liver are shown in Figure 2 and Figure 3, respectively. The results of oil-red O staining in muscle and liver are shown in Figure 4. The numbers of lipid droplets in muscle and liver were not significantly influenced by the different n-3 HUFA contents (*p* > 0.05).

### 3.6. Gene Expression related to Lipid Metabolism and Inflammatory Factors

**Figure 5:**
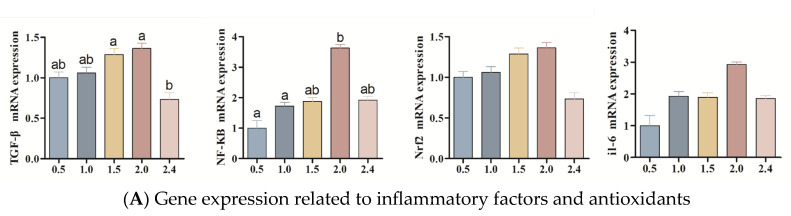

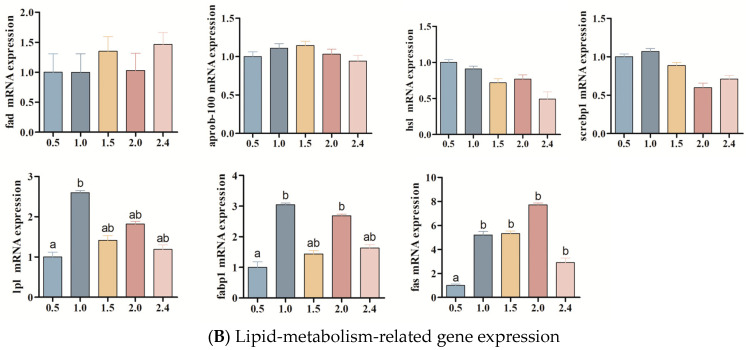
Effects of dietary n-3 HUFA on gene expression related to liver lipid metabolism, spleen inflammatory factors, and antioxidant levels in F2 generation female Yangtze sturgeon Acipenser dabryanus. Values are expressed as means ± SE (n = 6). Different letters indicate significant differences (*p* > 0.05).

The gene expression levels related to inflammatory factors, antioxidants, and lipid metabolism are presented in Figure 5A and Figure 5B, respectively. The expression levels of the transforming growth factor beta (TGF-β) in the spleens of fish fed diets containing 1.5% and 2.0% n-3 HUFA were significantly elevated compared to those fed a diet with 2.4% n-3 HUFA (*p* < 0.05). Additionally, no statistically significant differences were observed in TGF-β expression among fish fed diets with 0.0%, 1.0%, 1.5%, and 2.0% n-3 HUFA (*p* > 0.05). The expression of nuclear factor kappa-B (*nf*-κB) in the spleen of fish fed a diet containing 2.0% n-3 HUFA was significantly elevated compared to those fed diets with 0.5% and 1.0% n-3 HUFA (*p* < 0.05). The expression of the nuclear factor erythroid-2-related factor (*Nrf2*) and interleukin-6 (*Il*-*6*) in the spleen were not affected by the dietary n-3 HUFA contents (*p* > 0.05).

The expression of the fatty acid desaturase (*fad*), apolipoprotein b100 (*aprob 100*), hormone-sensitive lipase (*hsl*), and sterol regulator element-binding protein-1 (*srebp-1*) in the liver were not affected by the dietary n-3 HUFA diets (*p* > 0.05). The expression levels of lipoprotein lipase (*lpl*), fatty acid binding protein 1 (*fabp1*), and fatty acid synthase (*fas*) in the liver of fish fed a diet with 1.0% n-3 HUFA were significantly higher than in those fed a diet with 0.5% n-3 HUFA (*p* < 0.05). Furthermore, no statistically significant differences were observed among the groups of fish that were supplemented with various concentrations of n-3 HUFA in their diets (*p* > 0.05). 

### 3.7. Intestinal Microbiota Analyses

The alpha diversity, including the ACE, PD_whole tree, Richness, Chao1, Shannon, Simpson and Pielou, is presented in Table 8. The coverage for all five groups was 99.99%, indicating that the sequencing depth was adequate and accurately reflected the actual composition of the intestinal microbiota in Yangtze sturgeon. There was no significant difference between Shannon, Simpson and Pielou among the different groups (*p* > 0.05). The PD_whole tree, Richness, and Chao1 indices were elevated in fish fed diets with increased n-3 HUFA levels, particularly in those fed the 2.4% n-3 HUFA diet, compared to fish fed the 0.5% n-3 HUFA diet (*p* < 0.05).

**Figure 6:**
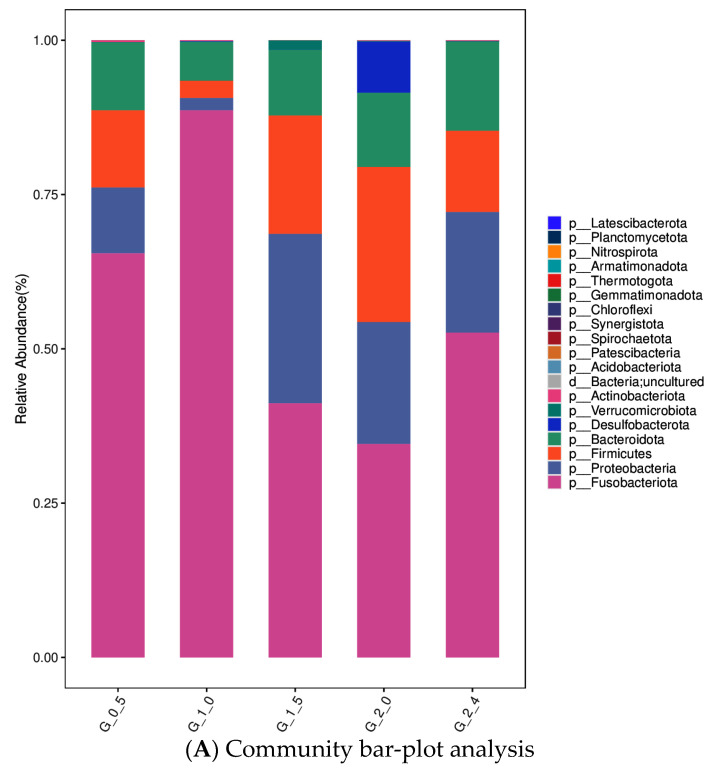

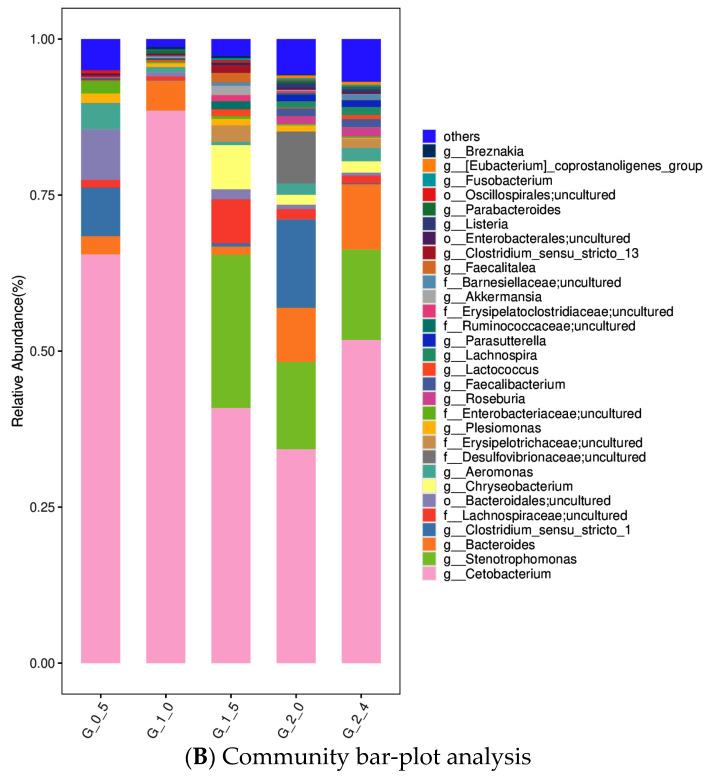
Intestinal microflora structure of each group at the phylum (**A**) and genus (**B**) levels in F_2_ generation female Yangtze sturgeon *Acipenser dabryanus* fed diets containing different levels of n-3 HUFA.

Furthermore, the intestinal microbiota of the different groups was investigated at both the phylum and genus levels (Figure 6). At the phylum level, the dominant phylum in the G_0.5 group was Fusobacteriota (65%), followed by Firmicutes (13%), Proteobacteria (11%), and Bacteroidota (11%); in the G_1.0 group, it was Fusobacteriota (89%), followed by Firmicutes (3%), Proteobacteria (2%), and Bacteroidota (6%); in the G_1.5 group, it was Fusobacteriota (41%), followed by Firmicutes (19%), Proteobacteria (27%), and Bacteroidota (11%); in the G_2.0 group, it was Fusobacteriota (35%), followed by Firmicutes (25%), Proteobacteria (20%), Desulfobacterota (8%), and Bacteroidota (12%); in the G_2.4 group, it was Fusobacteriota (53%), followed by Firmicutes (13%), Proteobacteria (20%), and Bacteroidota (14%) (Figure 6A). The results indicated that Fusobacteriota, Firmicutes, Bacteroidota, and Proteobacteria were the four most abundant bacterial phyla in the intestines of Yangtze sturgeon across the five groups. Based on the comparison of microbiota taxa at the genus level, the primary genera in the G_0.5 group was found to be *g*_*Cetobacterium* (65.47%), followed by *o*_*Bacteroidales* (8.1%), *g*_*Clostridium*_*sensu*_*stricto*_1 (7.82%), and *g*_*Aeromonas* (4.21%). In the G_1.0 group, it was *g*_*Cetobacterium* (88.54%), followed by *g*_*Bacteroides* (4.80%). In the G_1.5 group, it was *g*_*Cetobacterium* (40.86%), followed by *f*_*Lachnospiraceae* (7.05%), *g*_*Stenotrophomonas* (24.58%), and *g*_*Chryseobacterium* (7.07%). In the G_2.0 group, it was *g*_*Cetobacterium* (34.23%), followed by *g*_*Clostridium*_sensu_stricto_1 (14.19%), *g*_*Bacteroides* (8.68%), *g*_*Stenotrophomonas* (13.98%), and *f*_*Desulfovibrionaceae* (8.28%). In the G_2.4 group, it was *g*_*Cetobacterium* (51.79%), followed by *g*_*Bacteroides* (10.47%) and *g*_*Stenotrophomonas* (14.47%) (Figure 6B).

## 4. Discussion

Although the 10-month feeding period in this study did not yield significant differences in growth performance among various groups of Yangtze sturgeon, it was observed that using regression analysis, the present study showed that maximum weight gain would be attained at a dietary HUFAs content of 1.3%. This result is higher than those for larval flounder, *Paralichthys olivaceus*; juvenile starry flounder, *Platichthys stellatus*; and juvenile grass carp, *Ctenopharyngodon Idella* [19,38,39], and lower than that for larval flounder at the *Artemia* feeding stage [40]. The variation in the quantity of n-3 HUFA required can be attributed to the species and size of the fish. Excessively high levels of n-3 HUFA (2.0% or 2.4%) were detrimental to growth; similar phenomena were also observed in other studies [38,41]. The negative effects of excessive n-3 HUFA may be due to the disturbance of the membrane polar lipid caused by the excessive accumulations of EPA and DHA in tissues [38]. In previous studies, when n-3 HUFA was administered for 5 months, the growth performance of the moderate n-3 HUFA group was roughly similar to that of the low-level group, accounting for approximately 60% [28]. However, after extending the feeding period for another 5 months, this growth difference became increasingly apparent, with a weight gain difference of approximately 22% between the highest and lowest groups. Although there was no apparent deficiency of n-3 HUFA in the control group, it is speculated that this phenomenon can be attributed to two factors: Firstly, the basal diet included a certain quantity of n-3 HUFA, which partially met the nutritional requirements of the Yangtze sturgeon for this fatty acid. Secondly, the duration of the experiment may have been insufficient to fully manifest the symptoms of n-3 HUFA deficiency. In addition, some studies on sturgeon have also reported that the appropriate DHA-to-EPA ratio for EPA- and DHA-enriched diets can improve early growth and parental performance [24,25,26]. Some reports on freshwater fish have also shown that the addition of DHA or EPA and the enrichment of DHA with EPA in the diet have shown positive responses [19,42,43,44,45]. These results fully demonstrated that dietary supplementation with n-3 HUFA is indispensable in the specific phase, considering the results among different fish species. 

The regulation of n-3 HUFA in the diet did not cause significant deposition of crude lipid in tissues. However, it was observed that the crude lipid content in muscle, liver, and ovary tissues tended to increase with the elevation of n-3 HUFA in the diet, reaching a peak at 1.5% n-3 HUFA. Subsequently, there was a trend of decrease as the n-3 HUFA level in the diet further increased. This suggests that the addition of 1.5% n-3 HUFA in diet can serve as an important reference for regulating crude lipid in tissues, considering the importance of DHA and EPA to the fish diet, as well as the significance of fish-flesh lipid for human consumption. It is noteworthy that tissue fatty acids are regulated by n-3 HUFA, and the changes in most tissue fatty acids tend to be consistent with those in diet fatty acids. Dietary EPA and DHA were notably incorporated into the muscle and ovary, and similar results were also observed in juvenile black seabream [18]. To elucidate the underlying mechanism by which n-3 HUFA regulates hepatic lipid content, a comprehensive analysis of hepatic lipid-metabolism genes was conducted, focusing on three pivotal aspects: lipid synthesis, lipid catabolism, and lipid transport. The relative gene expression of *lpl*, *fas*, and *fabp1* appeared to show a similar tendency, with higher expression levels found in the supplementation with moderate n-3 HUFA diets, and lower values recorded in lower or higher dietary n-3 HUFA. These results clearly demonstrate that lipid metabolism is tightly regulated by the concentration of n-3 HUFA in the diet. Maintaining a dynamic equilibrium between lipid lipogenesis and lipolysis, the group with a high level of n-3 HUFA did not exhibit an elevated expression of fatty acid synthesis genes. On the contrary, the expression of fatty acid synthesis genes was higher in the group with a medium level of n-3 HUFA supplementation. Ultimately, there was no discernible difference in the crude lipid content of the liver, suggesting that lipid homeostasis is governed by these genes. The expression of the gene *fas* was significantly affected by n-3 HUFA in juvenile black seabream [18]; this result is consistent with our study. It is noteworthy that the expression trend of the *lpl* gene in the present results does not align with those of the aforementioned fish species [18], and neither does the present expression trend of the *fabp1* gene align with that determined in the golden pompano [17]. The reasons for these differences in research results may be mainly related to the subjects of this experiment. Freshwater fish and marine fish require different types of fatty acids, and thus their regulation by n-3 HUFA is not completely consistent.

Numerous effects of the n-3 fatty acids EPA and DHA on the functional responses of cells involved in inflammation and immunity have been described [46]. The spleen is a crucial immune organ. To investigate whether there are changes in pro-inflammatory and anti-inflammatory factors in Yangtze sturgeon fed with n-3 HUFA for 10 months, some related indicators were tested, which may provide important insights into the underlying mechanisms of fish immune responses. *Nf-κb* is the prototypical inflammatory transcription factor, which can mediate the production of pro-inflammatory cytokines (such as, *il-6*), thereby resulting in inflammation [17,47]. The *nrf2* (NF-E2-related factor 2) plays a crucial role in orchestrating cellular antioxidant defenses during oxidant stress conditions. As a pivotal transcription factor, Nrf2 recognizes antioxidant response elements (AREs), thereby initiating the transcription of genes encoding antioxidant enzymes such as SOD, CAT, and GST [48,49]. In this study, after feeding on n-3 HUFA diets, the gene expression of the pro-inflammatory factors *nf-κb* and *IL-6* peaked in the 2.0% n-3 HUFA group, whereas the gene expression of the anti-inflammatory factor *tgf-β* also attained its maximum in the same 2.0% n-3 HUFA group. This suggests that the balance between pro-inflammatory and anti-inflammatory factors in the body necessitates a dietary intake of at least 2% n-3 HUFA, and further study is needed to investigate the specific mechanisms involved.

The intestinal microbiome is one of the primary factors that exert a direct influence on the health and immunity of animals. Compared to mammals, the gut microbiome of fish remains relatively understudied. However, it is well-established that the composition of this microbiome is contingent upon the species of fish, environmental conditions, and, most importantly, diet [50,51]. In our study, indices such as ACE, PD_whole tree, Chao1, and Richness within α-diversity all demonstrated an increasing trend with the elevation of n-3 HUFA levels in the diet. While there were no significant differences observed among the Shannon, Simpson, and Pielou indices across various groups, there was also no declining trend in the group with high n-3 HUFA levels. The higher the abundance quantity of intestinal microbes, the more conducive it is for animals to deal with various environmental disturbances [52]. These results demonstrate that the current n-3 HUFA levels in the diet did not inhibit diversity levels, but instead contributed to the enhancement of certain α-diversity levels. From the perspective of the phylum distribution, Firmicutes, Proteobacteria, and Bacteroidota are the primary dominant phyla; this result shows partial similarity with those for hybrid sturgeon (*Acipenser baerii* ♂ × *A.schrenckii♀*), (*Acipenser baerii♀* × *A.schrenckii* ♂); Siberian sturgeon (*Acipenser baeri Brandt*); and Beluga sturgeon (*Huso huso*) [50,53,54], which may be an indication that those phyla are primary dominant among different species of sturgeon. Intriguingly, while Fusobacteriota is also a dominant phylum in the present study, it is not the dominant one in hybrid sturgeon, Siberian sturgeon, or Beluga sturgeon; it might be speculated that the cause of this phenomenon may be closely related to the feeding habits, diet composition, and farming environments of the fish. Meanwhile, changes in dietary n-3 HUFA also caused significant changes at the genus level; *Cetobacterium* was still the dominant genus in all groups, but showed a trend of decreasing with increasing dietary n-3 HUFA content. The research by Liu et al. [55] found that cellulose-degrading bacteria *Clostridium*, *Citrobacter*, and *Leptotrichia* were dominant in the herbivorous, while *Cetobacterium* and protease-producing bacteria *Halomonas* were dominant in the carnivorous. The aforementioned phenomenon strongly suggests that this bacterium could potentially be the predominant flora in the intestines of carnivorous fish. The feed formulation utilized in this trial was specifically tailored to align with the feeding habits of carnivorous fish. More importantly, it was found that the fish in the group with the highest abundance of *Cetobacterium* had the highest weight gain rate, and the trend in the abundance of *Cetobacterium* was relatively consistent with the trend in the fish weight gain rate. The *Cetobacterium* was the most abundant species in carnivorous channel catfish and largemouth bass [56]. These results suggested that bacterial species might play significant roles in their host’s digestion system.

## 5. Conclusions

After 10 months of feeding with n-3 HUFA, it was conclusively determined that incorporating 1.3% n-3 HUFA into the diet was suitable for the growth of stage II Yangtze sturgeon. The deposition of fatty acids in tissues was notably influenced by the fatty acid composition of the diet. Furthermore, the expression of certain genes related to lipid metabolism and inflammatory factors were regulated by n-3 HUFA. A positive correlation was observed between the diversity of gut microbiota and the dietary intake of n-3 HUFA. Specifically, the group fed with 1% n-3 HUFA exhibited the highest weight gain rate and the highest abundance of *Cetobacterium*. The aforementioned results offer valuable nutritional strategies for the artificial conservation of *Acipenser dabryanus*.

## Figures and Tables

**Table 1:** Formulation and ingredients of the experimental diet used for F_2_ generation female Yangtze sturgeon *Acipenser dabryanus* (%).

Ingredients	Dietary n-3 HUFA Level (% of Diet)				
	0.50	1.00	1.50	2.00	2.40
Fish meal	36.00	36.00	36.00	36.00	36.00
Soybean meal	16.00	16.00	16.00	16.00	16.00
Chicken meal	10.00	10.00	10.00	10.00	10.00
Squid meal	5.00	5.00	5.00	5.00	5.00
ARA-enriched oil	0.50	0.50	0.50	0.50	0.50
Soybean oil	5.00	4.50	4.00	3.50	3.00
DHA-enriched oil	0.00	0.25	0.50	0.75	1.00
EPA-enriched oil	0.00	0.25	0.50	0.75	1.00
Wheat flour	21.20	21.20	21.20	21.20	21.20
Choline chloride	0.20	0.20	0.20	0.20	0.20
Monocalcium phosphate	1.00	1.00	1.00	1.00	1.0
* Vitamin premix	1.00	1.00	1.00	1.00	1.00
* Mineral premix	2.00	2.00	2.00	2.00	2.00
Carboxymethylcellulose sodium	2.00	2.00	2.00	2.00	2.00
Mold inhibitor	0.05	0.05	0.05	0.05	0.05
Ethoxyquin	0.05	0.05	0.05	0.05	0.05
Proximate analysis (%)					
Moisture	6.60	7.41	7.99	7.62	6.90
Crude protein	42.39	42.33	42.79	43.33	41.87
Crude lipid	10.66	11.41	10.52	10.46	10.37
Ash	13.29	12.44	12.28	11.60	11.21

* Vitamin premixture provided the following per kg of diet: vitamin B_1_, 50 mg; vitamin B_2_, 200 mg; vitamin B_6_, 50 mg; vitamin B_12_, 20 mg; folic acid, 15 mg; vitamin C, 325 mg; calcium pantothenate, 400 mg; inositol, 1500 mg; *D*-biotin (2%), 5 mg; niacin, 750 mg; vitamin A, 2.5 mg; vitamin E, 160 mg; vitamin D3, 2.0 mg; vitamin K_3_, 20 mg. * Mineral premixture provided the following per kg of diet: Ca (H_2_PO4)_2_, 1800 mg; KH_2_PO_4_, 1350 mg; NaCl, 500 mg; MgSO·7H_2_O, 750 mg; NaH_2_PO_4_·2H_2_O, 650 mg; KI, 1.5 mg; CoSo_4_·6H_2_O, 2.5 mg; CuSo_4_·5H_2_O, 15 mg; ZnSO_4_·7H_2_O, 350 mg; FeSO_4_·7H_2_O, 1250 mg; MnSO_4_·4H_2_O, 80 mg; Na_2_SeO_3_, 6 mg.

**Table 2:** Growth performance and feed utilization of F2 generation female Yangtze sturgeon Acipenser dabryanus fed diets containing different levels of n-3 HUFA.

Parameter	Dietary n-3 HUFA Levels					*p*-Value
	0.50	1.00	1.50	2.00	2.40	
IBW/kg	3.43 ± 0.29	3.81 ± 0.27	3.52 ± 0.34	3.77 ± 0.19	3.43 ± 0.07	0.717
FBW/kg	6.82 ± 0.54	8.21 ± 0.47	7.36 ± 0.39	7.53 ± 0.39	6.59 ± 0.36	0.087
WGR/%	102.21± 14.33	117.77 ± 10.66	114.26 ± 10.95	102.63 ± 7.77	95.66 ± 12.51	0.633
CF/g/cm^3^	1.08 ± 0.05	1.16 ± 0.04	1.01 ± 0.03	1.12 ± 0.03	1.12 ± 0.07	0.313
VSI/%	4.58 ± 0.30	4.30 ± 0.22	4.32 ± 0.32	4.20 ± 0.20	4.44 ± 0.45	0.924
HSI/%	1.80 ± 0.04	1.79 ± 0.13	2.10 ± 0.23	1.89 ± 0.14	1.85 ± 0.13	0.569

Data for IBW, FBW, and WGR are presented as means ± SE (n = 15); data for CF, VSI, and HIS are presented as means ± SE (n = 6); data in the same line with different superscript letters indicate statistical significance (*p* < 0.05). IBW, initial body weight; FBW, final body weight; WGR, weight gain rate; CF, condition factor; HSI, hepatosomatic index; VSI, viscerosomatic index.

**Table 3:** Serum biochemical indexes of F_2_ generation female Yangtze sturgeon *Acipenser dabryanus* fed diets containing different levels of n-3 HUFA.

Parameter	Dietary n-3 HUFA Levels					*p*-Value
	0.00	1.00	1.50	2.00	2.40	
HDLC, mmol/L	0.24 ± 0.02	0.24 ± 0.01	0.29 ± 0.03	0.26 ± 0.01	0.26 ± 0.02	0.302
LDLC, mmol/L	2.15 ± 0.25	1.77 ± 0.05	2.28 ± 0.13	1.65 ± 0.22	1.82 ± 0.31	0.220
TG, mmol/L	6.48 ± 0.77 ^a^	4.51 ± 0.41 ^ab^	4.30 ± 0.49 ^abc^	2.26 ± 0.21 ^c^	3.75 ± 0.41 ^bc^	0.000
TCHO, mmol/L	2.81 ± 0.24	2.28 ± 0.11	2.83 ± 0.21	2.20 ± 0.23	2.48 ± 0.31	0.195

Data are presented as means ± SE (n = 6); data in the same line with different superscript letters indicate a statistical significance (*p* < 0.05). Abbreviations: HDLC, high-density lipoprotein cholesterol; LDLC, low-density lipoprotein cholesterol; TCHO, total cholesterol; TG, triglycerides.

**Table 4:** Liver, muscle, and ovary proximate compositions of F_2_ generation female Yangtze sturgeon *Acipenser dabryanus* fed diets containing different levels of n-3 HUFA (%).

Item	Dietary n-3 HUFA Levels					*p*-Value
	0.50	1.00	1.50	2.00	2.40	
Liver						
Moisture	53.01 ± 1.44	52.93 ± 3.27	50.62 ± 3.11	53.27 ± 4.13	52.47 ± 3.47	0.852
Crude protein	9.56 ± 0.72	9.01 ± 0.48	9.15 ± 0.25	8.67 ± 0.55	9.66 ± 0.38	0.670
Crude lipid	32.96 ± 1.85	29.31 ± 0.22	35.68 ± 2.25	34.66 ± 2.33	32.73 ± 2.18	0.398
Muscle						
Moisture	72.48 ± 3.15	79.62 ± 3.37	76.06 ± 10.33	72.43 ± 0.63	73.00 ± 1.33	0.399
Crude protein	20.21 ± 0.68	13.72 ± 1.64	15.62 ± 3.60	19.41 ± 0.19	19.81 ± 0.51	0.103
Crude lipid	5.66 ± 0.76	5.26 ± 0.19	7.16 ± 2.62	6.32 ± 0.35	5.53 ± 0.67	0.832
Ovary						
Moisture	31.81 ± 3.71	33.72 ± 1.63	29.17 ± 1.57	36.54 ± 8.43	35.63 ± 1.62	0.624
Crude protein	4.82 ± 0.12	5.75 ± 0.25	4.12 ± 0.31	4.63 ± 0.99	3.81 ± 0.05	0.193
Crude lipid	62.90 ± 3.73	59.62 ± 2.37	65.85 ± 1.78	56.78 ± 10.04	59.53 ± 1.21	0.593

Data are presented as means ± SE (n = 6), data in the same line with different superscript letters indicate a statistical significance (*p* < 0.05).

**Table 5:** Fatty acid profiles in muscle of F_2_ generation female Yangtze sturgeon *Acipenser dabryanus* fed diets containing different levels of n-3 HUFA (% total fatty acids).

Fatty Acid	Different Levels of n-3 HUFA					
	0.5	1.0	1.5	2.0	2.4	ANOVA (*p*)
C14:0	0.94 ± 0.01	0.85 ± 0.05	0.83 ± 0.04	0.85 ± 0.03	0.87 ± 0.05	0.471
C16:0	20.41 ± 0.40	20.26 ± 0.27	19.41 ± 0.09	19.09 ± 0.39	18.96 ± 0.36	0.066
C16:1	2.23 ± 0.09 ^a^	2.21 ± 0.09 ^a^	2.75 ± 0.08 ^b^	2.63 ± 0.04 ^b^	2.72 ± 0.04 ^b^	0.001
C17:0	0.13 ± 0.02 ^a^	0.14 ± 0.01 ^ab^	0.20 ± 0.01 ^ab^	0.22 ± 0.02 ^b^	0.21 ± 0.02 ^b^	0.009
C18:0	3.21 ± 0.12 ^a^	3.12 ± 0.17 ^ab^	2.68 ± 0.10 ^b^	2.90 ± 0.07 ^ab^	2.68 ± 0.03 ^b^	0.019
C18:1n9	33.72 ± 0.45	35.27 ± 1.29	34.16 ± 1.23	32.42 ±1.21	31.75 ± 1.63	0.337
C18:2n-6	24.14 ± 0.37 ^a^	22.03 ± 0.61 ^ab^	21.06 ± 0.36 ^b^	20.00 ± 0.28 ^bc^	18.28 ± 0.92 ^c^	0.000
C18:3n-6	1.23 ± 0.04 ^a^	0.92 ± 0.02 ^b^	0.92 ± 0.04 ^b^	0.89 ± 0.05 ^b^	0.76 ± 0.07 ^b^	0.001
C20:1	1.66 ± 0.04 ^a^	1.75 ± 0.07 ^a^	1.40 ± 0.04 ^a^	1.74 ± 0.21 ^a^	2.28 ± 0.07 ^b^	0.003
C18:3n-3	1.49 ± 0.03 ^ab^	1.59 ± 0.23 ^ab^	2.56 ± 0.03 ^c^	2.03 ± 0.16 ^bc^	1.29 ± 0.06 ^a^	0.000
C20:2	0.98 ± 0.08 ^a^	0.87 ± 0.07 ^ab^	0.85 ± 0.02 ^ab^	0.82 ± 0.04 ^ab^	0.66 ± 0.03 ^b^	0.021
C20:3n-6	0.78 ± 0.05 ^a^	0.65± 0.03 ^ab^	0.60 ± 0.02 ^b^	0.59 ± 0.03 ^b^	0.51 ± 0.03 ^b^	0.006
C20:4n-6 (ARA)	2.59 ± 0.07	2.36 ± 0.10	2.54 ± 0.16	2.77 ± 0.09	2.73 ± 0.12	0.163
C20:5n-3 (EPA)	1.48 ± 0.01 ^a^	2.42 ± 0.12 ^a^	3.98 ± 0.32 ^b^	5.08 ± 0.24 ^b^	6.95 ± 0.37 ^c^	0.000
C22:6n-3 (DHA)	4.02 ± 0.14 ^a^	4.69 ± 0.21 ^ab^	6.03 ± 0.52 ^bc^	7.93 ± 0.53 ^cd^	9.29 ± 0.52 ^d^	0.000

Data are presented as means ± SE (n = 3); data in the same line with different superscript letters indicate a statistical significance (*p* < 0.05). Abbreviations: ARA, arachidonic acid; DHA, docosahexaenoic acid; EPA, eicosapentaenoic acid.

**Table 6:** Fatty acid profiles in ovarian of F_2_ generation female Yangtze sturgeon *Acipenser dabryanus* fed diets containing different levels of n-3 HUFA (% total fatty acids).

Fatty Acid	Different Levels of n-3 HUFA					
	0.5	1.0	1.5	2.0	2.4	ANOVA (*p*)
C14:0	1.00 ± 0.04	0.98 ± 0.03	0.92 ± 0.01	0.98 ± 0.00	0.98 ± 0.03	0.367
C15:0	0.13 ± 0.00	0.13 ± 0.00	0.13 ± 0.00	0.14 ± 0.01	0.14± 0.00	0.336
C16:0	21.04 ± 0.37 ^a^	20.00 ± 0.09 ^ab^	20.29 ± 0.38 ^ab^	18.99 ± 0.45 ^b^	18.89 ± 0.33 ^b^	0.007
C16:1	2.33 ± 0.02	2.26 ± 0.03	2.43 ± 0.10	2.29 ± 0.05	2.39 ± 0.11	0.463
C17:0	0.14 ± 0.01	0.15 ± 0.01	0.13 ± 0.00	0.15 ± 0.01	0.14 ± 0.00	0.501
C18:0	3.07 ± 0.03 ^a^	3.03 ± 0.06 ^ab^	2.68 ± 0.04 ^bc^	2.90 ± 0.11 ^abc^	2.58 ± 0.11 ^c^	0.005
C18:1n9	34.66 ± 0.55	34.36 ± 0.81	33.86 ± 0.58	31.25 ± 1.07	30.69 ± 2.06	0.105
C18:2n-6	24.35 ± 0.44 ^a^	23.82 ± 0.40 ^ab^	21.12 ± 0.32 ^c^	21.44 ± 0.56 ^bc^	19.22 ± 0.83 ^c^	0.000
C18:3n-6	1.29 ± 0.11 ^a^	0.97 ± 0.05 ^ab^	0.66 ± 0.03 ^bc^	0.79 ± 0.06 ^bc^	0.51 ± 0.08 ^c^	0.000
C20:1	1.19 ± 0.04	1.28 ± 0.00	1.31 ± 0.03	1.32 ± 0.03	1.27± 0.08	0.326
C18:3n-3	2.16 ± 0.03 ^a^	2.06 ± 0.03 ^a^	1.82 ± 0.04 ^b^	1.75 ± 0.04 ^bc^	1.56 ± 0.07 ^c^	0.000
C20:2	0.90 ± 0.10	0.87 ± 0.01	0.77 ± 0.03	0.78 ± 0.06	0.65 ± 0.04	0.082
C20:3n-6	0.63 ± 0.07	0.60 ± 0.03	0.49 ± 0.02	0.53 ± 0.04	0.48 ± 0.07	0.208
C22:1	0.48 ± 0.06	0.47 ± 0.04	0.51 ± 0.02	0.50 ± 0.02	0.51 ± 0.03	0.934
C20:3n-3	0.26 ± 0.01 ^ab^	0.27 ± 0.00 ^a^	0.26 ± 0.00 ^ab^	0.25 ± 0.01 ^ab^	0.22 ± 0.01 ^b^	0.052
C20:4n-6 (ARA)	1.82 ± 0.02	1.89 ± 0.07	1.86 ± 0.02	1.98 ± 0.07	2.10 ± 0.26	0.561
C20:5n-3 (EPA)	1.28 ± 0.08 ^a^	2.36 ± 0.11 ^b^	4.30 ± 0.16 ^c^	5.74 ± 0.19 ^d^	7.66 ± 0.31 ^e^	0.000
C22:6n-3 (DHA)	3.23 ± 0.01 ^a^	4.47 ± 0.18 ^ab^	6.40 ± 0.34 ^bc^	8.16 ± 0.33 ^cd^	9.91 ± 0.97 ^d^	0.000

Data are presented as means ± SE (n = 3); data in the same line with different superscript letters indicate a statistical significance (*p* < 0.05). Abbreviations: ARA, arachidonic acid; DHA, docosahexaenoic acid; EPA, eicosapentaenoic acid.

**Table 7:** Fatty acid profiles in liver of F_2_ generation female Yangtze sturgeon *Acipenser dabryanus* fed diets containing different levels of n-3 HUFA (% total fatty acids).

Fatty Acid	Different Levels of n-3 HUFA					
	0.5	1.0	1.5	2.0	2.4	ANOVA (*p*)
C14:0	0.75 ± 0.02	0.58 ± 0.07	0.63 ± 0.06	0.78 ± 0.10	0.68 ± 0.16	0.603
C16:0	24.97 ± 1.06	24.79 ± 0.94	24.10 ± 0.77	23.75 ± 1.24	22.77 ± 2.16	0.774
C16:1	2.38 ± 0.03	2.58 ± 0.30	2.53 ± 0.32	2.82 ± 0.26	2.69 ± 0.22	0.773
C18:0	4.73 ± 0.14	4.36 ± 0.16	4.14 ± 0.45	3.63 ± 0.09	3.64 ± 0.49	0.139
C18:1n9	47.89 ± 1.22	52.32 ± 1.16	51.07 ± 1.77	47.28 ± 3.27	48.27 ± 6.26	0.782
C18:2n-6	11.09 ± 1.23	7.78 ± 1.31	9.63 ± 1.61	10.72 ± 2.18	10.58 ± 4.75	0.893
C18:3n-6	2.07 ± 0.43 ^a^	1.43 ± 0.20 ^ab^	1.00 ± 0.11 ^ab^	1.34 ± 0.29 ^ab^	0.65 ± 0.19 ^b^	0.037
C20:1	0.67 ± 0.06	0.79 ± 0.00	0.81 ± 0.12	0.91 ± 0.14	0.83 ± 0.19	0.791
C18:3n-3	0.97 ± 0.12	0.82 ± 0.07	0.90 ± 0.08	0.90 ± 0.11	0.87 ± 0.19	0.943
C20:2	0.67 ± 0.11	0.55 ± 0.07	0.60 ± 0.09	0.68 ± 0.16	0.53 ± 0.13	0.839
C20:3n-6	0.69 ± 0.19	0.52 ± 0.08	0.43 ± 0.05	0.57 ± 0.18	0.37 ± 0.14	0.570
C20:4n-6 (ARA)	1.03 ± 0.02	1.03 ± 0.10	0.84 ± 0.07	1.17 ± 0.19	1.05 ± 0.21	0.635
C20:5n-3 (EPA)	0.30 ± 0.00	0.42 ± 0.03	0.73 ± 0.14	1.25 ± 0.26	2.33 ± 1.10	0.101
C22:6n-3 (DHA)	1.16 ± 0.01	1.52 ±0.20	2.05 ± 0.36	3.55 ± 1.08	4.17 ± 1.84	0.203

Data are presented as means ± SE (n = 3); data in the same line with different superscript letters indicate a statistical significance (*p* < 0.05). Abbreviations: ARA, arachidonic acid; DHA, docosahexaenoic acid; EPA, eicosapentaenoic acid.

**Table 8:** Alpha diversity index of F_2_ generation female Yangtze sturgeon *Acipenser dabryanus* fed diets containing different levels of n-3 HUFA.

Parameter	Dietary n-3 HUFA Levels					*p* Value
	0.50	1.00	1.50	2.00	2.40	
Coverage	0.99 ± 0.00	0.99 ± 0.00	0.99 ± 0.00	0.99 ± 0.00	0.99 ± 0.00	0.053
ACE	95.74 ± 22.90 ^a^	103.11± 23.17 ^a^	111.54± 23.29 ^a^	156.25± 47.68 ^ab^	291.69 ± 54.40 ^b^	0.005
PD_whole tree	5.80 ± 1.46 ^a^	6.33 ± 0.95 ^a^	7.43 ± 1.17 ^a^	13.51 ± 3.59 ^ab^	16.59 ± 2.47 ^b^	0.004
Richness	91.33 ± 21.73 ^a^	94.67 ± 21.66 ^a^	106.00 ± 22.15 ^a^	149.00 ± 45.45 ^ab^	277.17 ± 51.76 ^b^	0.004
Chao1	95.94 ± 23.18 ^a^	102.95 ± 22.99 ^a^	109.15 ± 22.89 ^a^	155.53 ± 48.10 ^ab^	289.99 ± 53.90 ^b^	0.005
Shannon	1.59 ± 0.33	1.13 ± 0.29	1.64 ± 0.36	1.69 ± 0.53	2.14 ± 0.40	0.514
Simpson	0.60 ± 0.09	0.47 ± 0.08	0.58 ± 0.08	0.54 ± 0.11	0.63 ± 0.09	0.798
Pielou	0.35 ± 0.06	0.25 ± 0.05	0.36 ± 0.06	0.33 ± 0.08	0.37 ± 0.06	0.648

Data are presented as means ± SE (n = 6); data in the same line with different superscript letters indicate a statistical significance (*p* < 0.05).

## Data Availability

The data are presented within the article as well as in the Supplementary Materials.

## Supplementary Materials

The following supporting information can be downloaded at: https://www.mdpi.com/article/10.3390/ani14233523/s1, Table S1: Fatty acid composition of the experimental diets with different levels of n-3 HUFA (% total fatty acids); Table S2: Primer used for real-time PCR analysis.
