# Supplementary material for: The Influence of Dietary n-3 Highly Unsaturated Fatty Acids on Growth, Fatty Acid Profile, Lipid Metabolism, Inflammatory Response, and Intestinal Microflora in F2 Generation Female Yangtze Sturgeon (Acipenser dabryanus)
Source: Animals (Basel). 2024 Dec 5;14(23):3523. doi: 10.3390/ani14233523 (PMC11640325; doi:10.3390/ani14233523)
Supplement: Supplementary file 1 [file animals-14-03523-s001.zip › animals-3299316-supplementary.pdf]

Table S1. Fatty acid composition of the experimental diets with different levels of n-3 HUFA (% total fatty acids).

Fatty acid	Dietary n-3 HUFA levels				
	0.50	1.00	1.50	2.00	2.40
C14: 0	1.08	1.05	0.88	1.26	1.08
C15: 0	0.15	0.15	0.13	0.14	0.14
C16: 0	16.72	15.53	14.61	14.52	14.09
C16: 1	2.03	2.13	2.03	2.38	2.08
C17: 0	0.14	0.15	0.15	0.00	0.15
C17: 1	0.14	0.00	0.00	0.00	0.00
C18: 0	4.55	4.26	4.18	4.05	3.80
C18: 1n9	21.47	20.00	18.99	17.19	16.80
C18: 2n6	37.59	35.67	33.46	29.21	26.12
C20: 0	0.25	0.23	0.23	0.23	0.21
C18: 3n6	0.00	0.02	0.11	0.13	0.11
C20: 1	1.65	1.46	1.75	0.92	1.26
C18: 3n3	3.84	3.62	3.46	3.06	2.76
C20: 2	0.13	0.15	0.14	0.17	0.14
C22: 0	0.31	0.27	0.31	0.27	0.25
C20: 3n-6	0.33	0.34	0.36	0.35	0.35
C20: 4n-6 (ARA)	2.74	2.90	2.84	3.02	2.86
C20: 5 (EPA)	2.16	5.47	8.02	12.62	15.44
C24: 0	0.40	0.35	0.50	0.63	0.60
C24: 1	0.19	0.17	0.24	0.21	0.19
C22: 6n-3 (DHA)	4.02	5.98	7.51	9.55	11.46
n-3 HUFA	6.18	11.45	15.53	22.17	26.9

Abbreviations: ARA, arachidonic acid; DHA, docosahexaenoic acid; EPA, eicosapentaenoic acid; n-3 HUFA, n-3 highly unsaturated fatty acids (EPA+DHA).

Table S2. Primer used for real-time PCR analysis

Gene	Forward primer (5-3')	Reverse primer (5-3')	Annealing temperature (°C)
srebp-1	ATGCCAGTCAAAGGGGAGAA	CTGCATTGCCATTTTCAGCG	60/60
fabp1-1	AATGAGTCCAGTGCCTTTCG	CTCGGACAGTGAGGAGTTCC	60/64
aprob-100	TCAGCTTCTCTCGCAGTTCA	GCCCGTTCTCCTTTAGCAAG	60/62
lpl	ATTCCTACTTCGGCTGGTCC	TCTTGCGAACCTGGTCTTCT	62/60
fad	TTCCAGATTGAGCACCACCT	CCTCAGAGAGCTCACGATGT	60/62
fas	GCCACTTCAAACCCACCAAT	GTCACGACCTGAGAACTCCA	60/62
hsl	AAGGGGAGAGGCGAAAATGG	CGATGTTCTGCCGCTGAATG	62/62
tgf-β	TACTCATGTCAGTCCCGCAG	ATCCACTTCCAGCCCAGATC	62/62
nf- kb	ATTGTGAGAATGGACCGGGT	GCACAGACACAGGGTTCTTG	60/62
nrf2	TCTGATCTGAACGCACCCTT	TCTCGAAACCTTCAGCCACT	60/60
il-6	CTGGTTCGAGCTGCTCCTAT	GCAGCCACTCGGCTAAAGAT	62/62

Abbreviations: *srebp-1*, sterol regulator element-binding protein-1; *fabp1*, fatty acid binding protein 1; *aprob-100*, apolipoprotein b 100; *lpl*, lipoprotein lipase; *fad*, fatty acid desaturase; *fas*, fatty acid synthase; *hsl*, hormone-sensitive lipase; *tgf-β*, transforming growth factor beta; *nf- kb*, nuclear factor kappa b; *nrf2*, nuclear factor erythroid-2 related factor; *il-6*, interleukin-6.
